# Impact of Immersive Virtual Reality During Outpatient Sedation‐Free Colonoscopy: A Randomized Prospective Controlled Study

**DOI:** 10.1002/hsr2.70563

**Published:** 2025-04-06

**Authors:** Myriam Ayari, Sameh Riahi, Mohamed Hedi Douggui, Taieb Jomni

**Affiliations:** ^1^ Department of Gastroenterology Internal Security Forces Hospital La Marsa La Marsa Tunisia; ^2^ University of Tunis El Manar Tunis Tunisia

**Keywords:** anxiety, colonoscopy, comfort, immersion, pain, virtual reality

## Abstract

**Background and Aims:**

Colonoscopy is the gold standard for accurate exploration of the colon. Thus, it must be performed as efficiently as possible. The patient's tolerance considerably affects the quality of sedation free examinations. Pharmacological sedation can solve this issue; however, it can expose to significant adverse events. The aim of this study was to evaluate the impact of immersive virtual reality (VR) during sedation‐free colonoscopy.

**Methods:**

We conducted a prospective controlled study including outpatients presenting for unsedated colonoscopy. Patients were randomized into Group 1: colonoscopy with VR headset and Group 2: without intervention. Anxiety, comfort, and pain were respectively evaluated by State‐Trait Anxiety inventory (STAI), Gloucester scale and the verbal rating scale (VRS).

**Results:**

In total, 63 patients were included: intervention group G1 (*n* = 33) and control group G2 (*n* = 30). A slightly lower time to caecal intubation was noted in the intervention group without significant difference (G1 = 19 min vs. G2 = 26 min, *p* = 0.07). Patients with VR mask expressed lower levels of post‐procedural anxiety than those in the control group (mean STAI G1 = 47 vs. G2 = 53, *p* < 0.01) and a significant decrease in the STAI score compared to pre‐endoscopy values (8 vs. 4 points, *p* < 0.01). The per‐procedural pain assessed by VRS was significantly lower in the patients using VR (Mean G1 = 0.44 vs. G2 = 1.32, *p* < 0.01). Moreover, endoscopic examination was found to be more comfortable with VR based on the Gloucester scale *p* < 0.01.

**Conclusions:**

Immersive VR technology is a promising, noninvasive and well‐accepted tool for improving tolerance by reducing colonoscopy induced pain and anxiety allowing an optimized examination.

## 1 Introduction

Colonoscopy is the gold standard for the prevention, screening and diagnosis of colorectal lesions. Different guidelines have drawn up recommendations on quality indicators for colonoscopy. Indeed, optimal examination aim to reduce the rate of interval colorectal cancer, which in more than half of all cases is related to lesions missed at colonoscopy [1]. Thus, effort must, therefore, be made to ensure a high‐quality examination, guaranteeing safety and comfort, while minimizing the risk of missing lesions. However, during unsedated lower gastrointestinal endoscopy, patient's tolerance considerably affects the quality of the examination and exposes to a high rate of interruption. Moreover, the pain and anxiety induced by a colonoscopy performed without anesthesia could make the patient reluctant to undergo subsequent examinations [2]. Pharmacological sedation can solve this issue. Nevertheless and despite the nonnegligent added cost [3], it can expose in some cases to an increase of adverse events [4]. Moreover, anesthesia is not always available leading to postponed endoscopic procedure. In this context, nonpharmacological interventions such as preprocedural information [5], water exchange colonoscopy [6], distraction [7], complementary medicine [8], and colonoscope technology [9] are being newly used to reduce anxiety and pain. Immersive virtual reality (VR) refers to a relatively new technology that simulates a three‐dimensional, computer‐generated environment. This technology can transport users to virtual worlds or simulated environments, providing experiences that feel realistic. Application of immersive VR in medical field have interested specially pediatric population and was used in different procedure including venipuncture [10], preoperative anxiety [11], acute and chronic pain [12]. Substantial effect sizes suggest that VR serves as a highly effective distraction intervention for alleviating pain and anxiety among pediatric patients undergoing diverse medical procedures [13]. Recently, a review analyzing multiple databases and covering 118 studies concluded that VR is a feasible, safe, and effective approach in reducing psychological distress during colonoscopy [14]. The aim of our study was to investigate the impact of intraprocedural immersive VR combining visual and auditory distraction in the improvement of the tolerance and the progress during unsedated colonoscopy in Tunisian patients.

## 2 Patients and Methods

We conducted a prospective controlled study from February to April 2023. We have included outpatients aged over 18 years presenting to the endoscopy unit for scheduled unsedated colonoscopy for any indications. Noninclusion criteria were patients with severe visual and/or auditory impairment, severe psychiatric disorders, dementia, cognitive impairment, and epilepsy. After randomization, patient with incomplete colonoscopy or refusing to continue the experience during the examination were excluded.

Study design: Patients were randomized in a 1:1 ratio to either intervention or control group using a randomizer online platform. The number of groups was set to two representing the intervention group G1 (colonoscopy with VR headset) and the control group G2 (colonoscopy without VR headset). The randomization was set to equal allocation to ensure an even distribution of participants between the two groups. After initiating the randomization process, participants were assigned randomly to one of the two groups.

The material used was a VR headset from the brand VR box, compatible with Apple and Android smartphones and provided with a front tray and a fully adjustable headband. A smartphone was placed on the tray. The VR box headset was then placed on the patient's head. Adjustment knobs were used as well as head strap to regulate the focus and to make a comfortable fit. Finally, the VR content was played on the smartphone. The video content displayed on the hardware was made of several clips showing nature scenes. The audio content consisting in relaxing music was adapted to allow optimal communication with the patient. The adequate positioning of VR glasses during the entire procedure was assured. The devices were disinfected and cleaned after each use to maintain clear vision. All endoscopies were performed by experienced endoscopists. The colon was inspected with an endoscope using air insufflation without CO_2_. Scope‐guide functionality was not available.

Anxiety was assessed by the State‐Trait Anxiety Inventory (STAI) which is a self‐report questionnaire consisting of separate scales to measure state and trait anxiety. It is the most widely used tools for assessing anxiety in both clinical and research settings and Arabic version of the STAI was validated [15]. The 20‐item of the score is ranging from 20 (absence of anxiety) to 80 (high anxiety). State Anxiety scale indicate the intensity of anxiety symptoms experienced in response to a specific situation or context at the time of assessment. Higher scores suggest greater levels of temporary distress or apprehension. Anxiety scale reflects an individual's general predisposition to experience anxiety across various situations. Higher scores suggest a greater tendency to perceive situations as threatening and to respond with elevated levels of anxiety.

Comfort during endoscopy was assessed by the 5‐point modified Gloucester Comfort Scale ranging from 1 (no discomfort) to 5 (extreme discomfort) [16]. Pain was evaluated by the verbal rating scale (VRS) where 0 correspond to no pain and 3 to extreme pain [17]. Patients were asked to assess the degree of pain they experienced during the procedure according to the VRS 10 min after the colonoscopy was ended. No preoperative analgesic therapy (oral or gas mixture relieving pain) was used.

### 2.1 Outcome Measures

The primary outcome was to investigate the effectiveness of immersive VR masks in reducing anxiety, pain and discomfort and enhancing patient experience during colonoscopy. Secondary outcome was to assess the impact VR over duration of caecal intubation.

### 2.2 Data Collection

Data collection was performed 15 min before the procedure and 10 min after and carried out by a second physician who was present throughout the entire procedure.

For each patient, the following data were collected:

‐ Baseline demographic and clinical data.
‐ Medical history of abdominal/pelvic surgery and diverticular disease was checked for each patient.
‐ Pre‐procedure anxiety assessment by STAI.
‐ Discomfort and pain levels during colonoscopy evaluated by Gloucester scale and VRS respectively.
‐ Time to caecal intubation: For patients who underwent polypectomy, the time spent on this specific procedure was measured and subtracted from the total period of caecal intubation.
‐ Degree of colonic preparation assessed by the Boston score.
‐ Postprocedure anxiety assessment by STAI.

### 2.3 Statistical Analysis

The sample size was determined using an online sample size calculator (BiostatTGV). Calculations were made based on the assumption that study power (1 − *β*) is 90%, type I error (alpha) probability is 0.05 and effect size calculated as *f* = 0.55. Sample calculated for each group was 30. We estimated the total number of patients to be recruited at 74 to take into account excluded patients. Statistical analysis was performed using SPSS version 22 (International Business Machines Corporation, Armonk, New York, United States). Data were expressed as either mean ± standard deviation (SD) for continuous variables or frequencies (percentage) for categorical variables. Groups were compared using the chi square test for categorical variables and Student's *t*‐test for quantitative variables. All comparisons attained statistical significance at *p* < 0.05.

### 2.4 Informed Consent

Written and verbal informed consent was obtained from the participants.

### 2.5 Ethical Approval

The study was approved by the local ethical committee of La Marsa Internal Security Forces Hospital. Approval number: 10/2023.

## 3 Results

**Figure 1:**
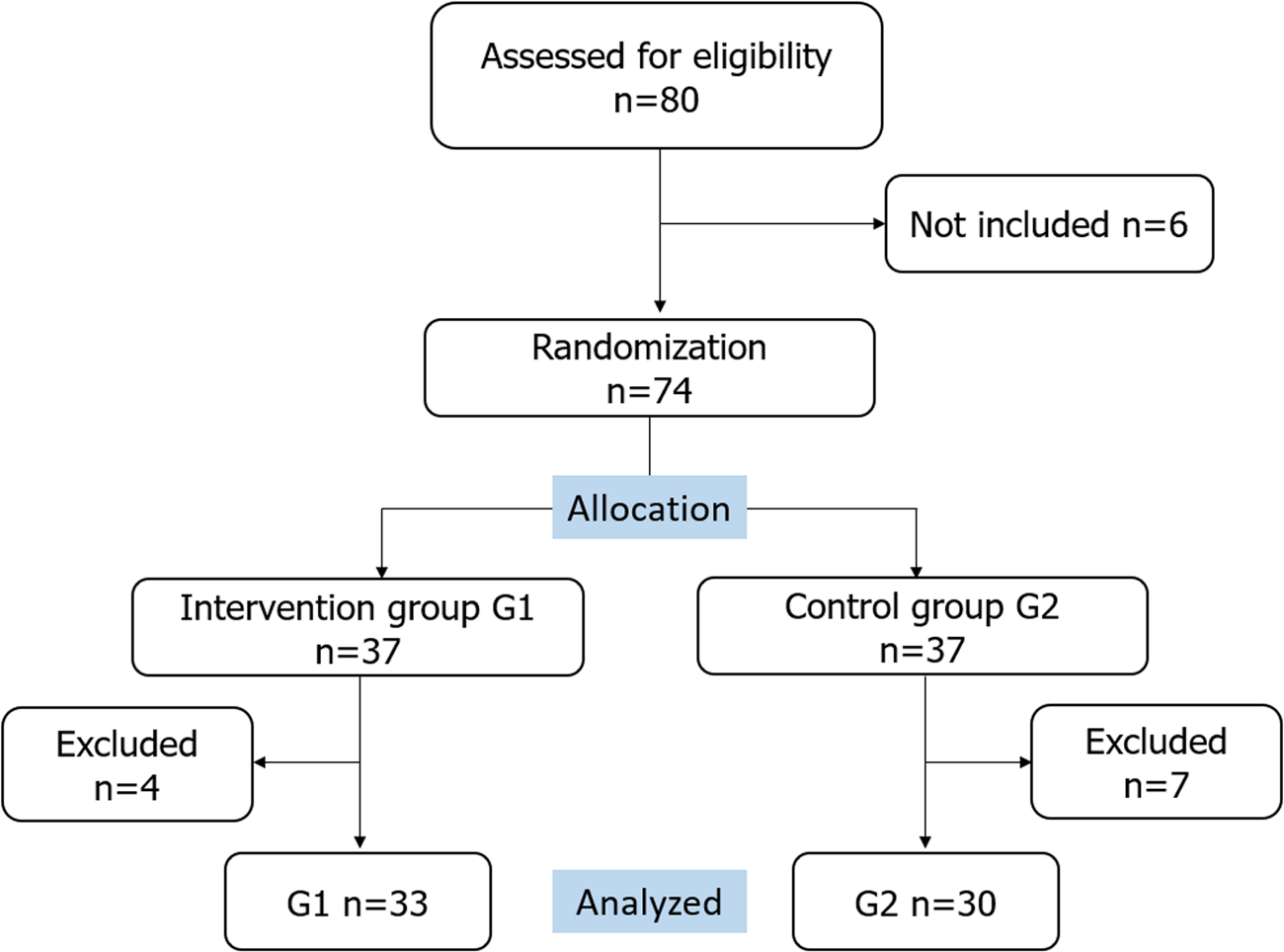
CONSORT flow diagram of the study.

In total, 63 patients were included in the final analysis: intervention group G1 (*n* = 33) and control group G2 (*n* = 30). The flowchart of the study is showed in Figure 1. There were four patients who removed their masks within the first 3 min as the procedure was felt extremely painful and they were unable to complete the procedure. The mean age was of 57 years with a sex‐ratio M/F of 1.25. No patient encountered a technical problem with the equipment used and no adverse events occurred during the immersive experience. Four patients were not satisfied with the resolution of the videos and 8 patients have expressed their preference to choose the content themselves.

### 3.1 Baseline Characteristics

When comparing baseline characteristics of the two groups, no significant differences were observed regarding gender, age, body mass index (BMI), history of abdominal surgery, or Boston preparation score (Table 1).

**Table 1:** Distribution of the baseline characteristics of the two groups.

	Virtual reality group (*n* = 33)	Control group (*n* = 30)	*p*‐Value
Age	53.74 ± 14.75	58.09 ± 14.43	0.23*
Gender			
Male	18 (54.5%)	17 (56.6%)	0.26**
Female	15 (45.4%)	13 (43.3%)	
History of abdominal surgery	8 (24.2%)	7 (21.2%)	0.87**
Body mass index	28.70 ± 4.43	27.6 ± 4.04	0.64*
Boston score	5.85 ± 0.78	6.09 ± 0.94	0.27*

* Student's *t*‐test.

** Chi square test.

### 3.2 Primary Outcome: Anxiety, Pain, and Comfort

**Figure 2:**
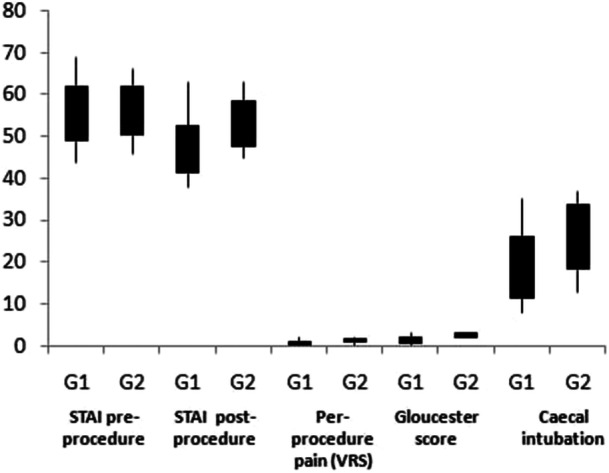
A graph comparing outcomes between the two groups. STAI, State‐Trait Anxiety Inventory; VRS, verbal rating scale.

Regarding anxiety, there was no significant difference between pre‐procedure STAI scores between the two groups (Table 2 and Figure 2). However, patients in the intervention group expressed significantly lower levels of postprocedural anxiety than those in the control group (mean STAI G1: 47 vs. G2: 53, *p* < 0.001). Moreover, patients with VR mask manifested a substantial reduction in points change of anxiety state levels when comparing before and after colonoscopies STAI values (reduction of 8 points vs. 4 points, *p* < 0.01). The per‐procedural pain assessed by EVS was significantly lower in the patients using VR (mean G1:0.44 vs. G2:1.32, *p* < 0.01). In addition, endoscopic examination was found to be more comfortable with VR based on the Gloucester scale *p* < 0.01 (Table 2, Figure 2).

**Table 2:** Comparison of anxiety, pain, comfort, and procedure duration between control and experimental groups.

	Virtual reality (*n* = 33)	Control group (*n* = 30)	*t*‐Value*	*p*‐Value*
STAI				
Pre‐procedure	55.5 ± 6.54	56.12 ± 5.73	0.413	0.68
Postprocedure	47 ± 5.45	53 ± 5.44	3.866	<0.001
Per‐procedure pain (VRS)	0.44 ± 0.56	1.32 ± 0.50	7.448	<0.001
Gloucester score	1.29 ± 0.629	2.48 ± 0.508	8.512	<0.001
Caecal intubation	19.18 ± 6.842	26.24 ± 7.673	2.854	0.07

Abbreviations: STAI, State‐Trait Anxiety Inventory; VRS, verbal rating scale.

* Student's *t*‐test.

### 3.3 Secondary Outcome: Procedure Time

In terms of colonoscopy duration, a slightly lower time to caecal intubation was noted in the intervention group without significant difference (Table 2 and Figure 2).

## 4 Discussion

Sedation‐free colonoscopy is associated with anxiety and pain in an interdependent manner. Pharmacological sedation allows overcoming these issues, however at the cost of other drawbacks. Most important to list are the interruption of daily activities, the adverse effects of anesthetic drugs, the significant cost of the procedure, the unavailability of suitable platforms and the long waiting time for appointments. Therefore, research directions pursue raising sensitivity of currently available alternatives of pharmacological sedation. VR has been under the spotlight and was acknowledged as a holistic care device complementary to established approaches at various tiers of healthcare. It is rapidly transforming healthcare delivery and redefining providers' approach towards patient care. The evolution of VR technology empowers healthcare professionals to broaden treatment horizons and offer pioneering solutions in patient management.

Continuous research efforts are targeting a wider uptake of VR in various medical fields. In fact, studies reported that VR could alleviate anxiety in breast cancer patients undergoing chemotherapy [18]. Moreover, VR has proven its efficiency as a relatively low‐cost pain therapy in the management of acute and chronic pain. VR analgesia extended into multiple areas such as burn care therapy [19], dental treatment [20], early stages of labour [21], phantom limb's treatment [22, 23], spinal injury [24], chronic pain [25], breast cancer patients after mastectomy [26], subacomial impingement syndrome, and scapular dyskinesis [27] as well as chronic musculoskeletal disorders [28]. Given the importance of improving patient comfort and compliance during endoscopy, there is a need to explore innovative technologies in this area, with VR emerging as a particularly promising tool. Here, we report the first Tunisian randomized study investigating the impact of VR in the gastroenterology field. In our study, VR significantly improved tolerance by reducing pain and anxiety leading a more comfortable examination. The clear process explaining the effect of VR is not completely elucidated. The main mechanisms of VR analgesia are distraction, focus‐shifting and skill‐building. In the literature, the immersive distraction provided by VR was reported to induce a pain‐relieving effect and has been utilized as a complementary tool to manage chronic pain [29]. In fact, pain demands attention, by creating overpowering diversion through an immersive experience, a more slowly response to pain signals will be generated by disrupting these signals as it is difficult to perceive solicitations outside the attention filed [30]. Indeed, the solicitation of multisensory information both visual and auditory, during endoscopy with VR mask, would distract the mind from processing pain induced during the examination [31]. Also, VR provides an isolation from external medical setting eliciting in many patients significant stress related to the apprehension of the slightest medical remarks during the exam. This has been remarkably noted in patients with somatoform complaints, which refers to physically unexplained complaints. In fact these patients, are often prone to stress and in particular less able to cope successfully with unsedated colonoscopy [32]. Focus‐shifting consists of tracking multiple objectives that can influence interaction priorities and redirecting patient attention from one virtual goal to another [33]. Engaging children in VR games tracking moving virtual targets, has proven effective during venipuncture [34]. Finally, skill‐building is the most complicated mechanism to implement since it must be actively pursued by the practitioner. For instance, VR applications were used to instruct patients on how to better control their respiration during painful procedures [35]. In the current study, patients receiving the VR headset combining visual and auditory distraction were found to be significantly less painful, less anxious and more comfortable. Firstly, research have studied the effect of visual and/or auditory stimulation and have found it reduced pain during endoscopy [36, 37]. In the literature, studies investigating the use of VR during endoscopy are scare especially without sedation. In a prospective study including 19 patients and using VR glasses without audio content, there were no difference regarding comfort, pain, anxiety, in both groups. However, patients in VR group described a pleasant distracting effect [38]. Auditory isolation appears to be a major component in achieving the sedative effect of this technique since it implies otherwise focusing the patient's attention toward the same stressful procedure. A recent study including 120 patients and combining auditive with visual distraction aligned with our results [39]. In fact, a significant lower median pain score was reported in the VR group compared to the control group (*p* < 0.001) [39]. A customized controlled study conducted with 60 patients showed a statistically significant difference between the trait anxiety scores (*p* < 0.001) and pain scores (*p* < 0.03) during the procedure between VR group and control group [40]. When the use of VR during colonoscopy seems to be effective, its implementation before the procedure for the education of patients seems, also, improving the quality of bowel preparation and to alleviate anxiety before the exam [41, 42, 43]. The results of our study were consistent with those of another recent parallel randomized controlled open‐label trial involving 50 patients in terms of alleviating pain and procedural discomfort through VR [44].

The present results should be interpreted in light of some limitations. The beneficial effects of VR were not assessed through objective measures like heart rate, blood pressure. In fact it has been showed, that VR has resulted in a significant reduction in heart rate and skin conductance 5 min after the insertion of the colonoscope [39]. In our study, participants were not able to test the VR device before the endoscopy. A prior exposure may have been useful to better explain the concept of the technique.

## 5 Conclusion

In summary, our study showed a growing evidence of VR effectiveness in reducing anxiety and pain during colonoscopy, giving it a great potential to improve healthcare delivery and patient well‐being. Therefore, VR may be a useful alternative to pharmacological sedation in selected patients. These results are encouraging to enable patients choosing VR during free‐sedation colonoscopy. With ongoing technological advancements, the increasing affordability and quality of portable VR headsets are further arguments in favor of a more widespread use of this promising technique. However, some aspects are to take in consideration to improve the efficiency of VR. For instance, making VR a personalized experience tailored to personal differences and preferences, which will improve the sense of virtual presence perceived by the patient. The use of high‐end equipment is also very important to achieve this goal. Finally, larger studies are required to confirm our findings.

## Author Contributions

**Myriam Ayari:** conceptualization, investigation, writing–original draft, methodology, validation, supervision. **Sameh Riahi:** investigation, visualization, data curation, resources, formal analysis. **Mohamed Hedi Douggui:** writing–review and editing, supervision. **Taieb Jomni:** writing–review and editing, supervision.

## Conflicts of Interest

The authors declare no conflicts of interest.

## Transparency Statement

The lead author Myriam Ayari affirms that this manuscript is an honest, accurate, and transparent account of the study being reported; that no important aspects of the study have been omitted; and that any discrepancies from the study as planned (and, if relevant, registered) have been explained.

## Data Availability

The data that support the findings of this study are available from the corresponding author upon reasonable request.
